# Mechanism of inhibition of taurolithocholate‐induced retrieval of plasma membrane MRP2 by cyclic AMP and tauroursodeoxycholate

**DOI:** 10.14814/phy2.13529

**Published:** 2017-11-30

**Authors:** Se Won Park, Cynthia R. L. Webster, Mohammed S. Anwer

**Affiliations:** ^1^ Department of Biomedical Sciences Cummings School of Veterinary Medicine at Tufts University 200 Westboro Road North Grafton Massachusetts USA; ^2^ Department of Clinical Sciences Cummings School of Veterinary Medicine at Tufts University 200 Westboro Road North Grafton Massachusetts USA

**Keywords:** Cholestasis, HuH‐NTCP cells, MARCKS phosphorylation, PKC*ε*, rat hepatocytes

## Abstract

Taurolithocholate (TLC) produces cholestasis by inhibiting biliary solute secretion in part by retrieving MRP2 from the plasma membrane (PM). Tauroursodeoxycholate (TUDC) and cAMP reverse TLC‐induced cholestasis by inhibiting TLC‐induced retrieval of MRP2. However, cellular mechanisms for this reversal are incompletely understood. Recently, we reported that TLC decreases PM‐MRP2 by activating PKCε followed by phosphorylation of myristoylated alanine‐rich C kinase substrate (MARCKS). Thus, cAMP and TUDC may reverse TLC‐induced cholestasis by inhibiting the TLC/PKC *ε*/MARCKS phosphorylation pathway. We tested this hypothesis by determining whether TUDC and/or cAMP inhibit TLC‐induced activation of PKCε and phosphorylation of MARCKS. Studies were conducted in HuH‐NTCP cell line and rat hepatocytes. Activation of PKC *ε* was determined from the translocation of PKC *ε* to PM using a biotinylation method. Phosphorylation of MARCKS was determined by immunoblotting with a phospho‐MARCKS antibody. TLC, but not cAMP and TUDC, activated PKC *ε* and increased MARCKS phosphorylation in HuH‐NTCP as well in rat hepatocytes. Treatment with TUDC or cAMP inhibited TLC‐induced activation of PKCε and increases in MARCKS phosphorylation in both cell types. Based on these results, we conclude that the reversal of TLC‐induced cholestasis by cAMP and TUDC involves, at least in part, inhibition of TLC‐mediated activation of the PKCε/MARCKS phosphorylation pathway.

## Introduction

Transhepatic solute transport provides the osmotic driving force for canalicular bile formation. Cholestasis accompanying many liver diseases (Maillette de Buy and Beuers, 2010; Stapelbroek et al. 2010) results from inadequate solute transport across hepatocytes. It is now well established that transporters involved in bile formation undergo transcriptional as well as posttranslational regulations (Chiang 2013; Halilbasic et al. 2013), and the latter involves short‐term changes in the plasma membrane (PM) localization of these transporters (Anwer 2004; Crocenzi et al. 2012; Boyer 2013) allowing for rapid changes in bile formation. For example, cholestatic agents, such as taurolithocholate (TLC) and estradiol17*β*‐d‐glucuronide (E17G), inhibit biliary solute secretion in part by retrieving multidrug resistance‐associated protein 2 (MRP2; *ABCC2*) from the PM (Beuers et al. 2001; Mottino et al. 2002). Choleretic agents, such as tauroursodeoxycholate (TUDC) and cAMP, on the other hand, insert MRP2 (Roelofsen et al. 1998; Beuers et al. 2001; Schonhoff et al. 2008) into the PM. TUDC and cAMP also reverse cholestatic effect of TLC by inhibiting TLC‐induced retrieval of MRP2 (Beuers et al. 2001; Park et al. 2014; Miszczuk et al. 2015) and cAMP can reverse E17G‐induced retrieval of MRP2 (Mottino et al. 2002). Cellular mechanisms by which choleretic agents, such as cAMP and TUDC, reverse the effects of TLC on PM localization of MRP2 are incompletely understood.

It is becoming evident that the PM localization of MRP2 is a highly regulated process and a number of signaling pathways have been proposed (Anwer 2014). Studies have suggested that PKCε is involved in the retrieval of MRP2 by cholestatic agents (Beuers et al. 2003; Kubitz et al. 2004a; Crocenzi et al. 2008; Schonhoff et al. 2009), while PKCδ and p38 MAPK are involved in the insertion of MRP2 to PM by choleretic agents (Kurz et al. 2001; Kubitz et al. 2004b; Park et al. 2012). Cellular mechanisms involved in the reversal of these cholestatic effects have also been explored. Thus, the anticholestatic effects of glucagon and salbutamol against E17G‐induced retrieval of MRP2 may involve a protein kinase A (PKA) and a cAMP‐regulated guanine nucleotide exchange factor (EPAC)/mitogen‐activated protein kinases kinase (MEK) pathway, respectively (Zucchetti et al. 2011). A cooperative posttranslational PKCα/PKA‐dependent mechanism has been proposed for the reversal of TLC‐induced cholestasis by TUDC (Wimmer et al. 2008). Our recent study (Schonhoff et al. 2013) in a human hepatoma cell line (HuH‐NTCP) revealed that TLC decreased PM‐MRP2 by activating PKCε followed by phosphorylation of myristoylated alanine‐rich C kinase substrate (MARCKS), a PKC substrate, and an actin‐binding protein (Hartwig et al. 1992; Fujise et al. 1994). This study (Schonhoff et al. 2013) led us to the hypothesis that cAMP and TUDC may also reverse TLC‐induced retrieval of PM‐MRP2 by inhibiting TLC‐induced activation of PKCε/MARCKS phosphorylation pathway.

The aim of the present study was to test the above mentioned hypothesis by determining whether TUDC and/or cAMP inhibit TLC‐induced activation of PKCε and phosphorylation of MARCKS. Studies were conducted in rat hepatocytes as well as in HuH‐NTCP cells to evaluate any species and cell type (primary vs. hepatoma)‐dependent effects. Results of our studies in both cell types are consistent with the proposed hypothesis.

## Materials and Methods

### Materials

8‐(4‐Chlorophenylthio)‐cAMP (CPT‐cAMP), TUDC, and TLC were purchased from Sigma‐Aldrich (St. Louis, MO). Commercial sources of other antibodies were Cell Signaling Technology (Phospho‐MARCKS and PKC*ε*), Calbiochem (actin), and BD Transduction Laboratories (E‐Cadherin). These antibodies react with both human and rat proteins. Sulfosuccinimidyl‐6‐(biotin‐amido)‐hexanoate (Sulfo‐NHS‐LC‐Biotin) was purchased from Pierce (Rockford, IL). Streptavidin beads were purchased from Novagen (Madison, WI). HuH‐NTCP cells (HuH7 cells stably transfected with human NTCP) were generously provided by Gores (Higuchi et al. 2001).

### Rat hepatocytes

Rat hepatocytes were isolated from male Wistar rats (200–250 g) and cultured as described previously (Johnston et al. 2011). Male Wistar rats were obtained from Charles River Laboratories and the protocol for harvesting livers was approved by the Institutional Animal Care and Use Committee.

### Human cell line

HuH‐NTCP cells were grown in Eagle's minimum essential medium supplemented with 10% FCS, 1.2 g/L G418 100,000 units/L penicillin, 100 mg/L streptomycin, and 25 *μ*g/mL amphotericin B at 37°C with 5% CO_2_ as described previously (Schonhoff et al. 2013). After culturing, the medium was changed to serum‐free DMEM for 3 h and cells were then treated with or without CPT‐cAMP, TUDC, or TLC. Since NTCP transports conjugated bile acid into hepatocytes (Anwer and Stieger 2013), HuH‐NTCP cells were used to assure intracellular effects in this hepatoma cell line.

### Plasma membrane PKC*ε*

A cell surface protein biotinylation method as described previously by us (Webster and Anwer 1999; Webster et al. 2000; Schonhoff et al. 2008; Park et al. 2012) was used to assess PKC*ε* translocation to plasma membranes. A cell surface biotinylation method is shown to be a better measure of PM proteins and produces highly pure cell surface proteins (Elia 2012). Briefly, following various treatments, cell surface proteins were biotinylated by exposing hepatocytes to sulfo‐NHS‐LC‐Biotin followed by preparation of a whole cell lysate. Biotinylated proteins were isolated using streptavidin–agarose beads and then subjected to immunoblot analysis to determine plasma membrane PKC*ε* and E‐cadherin. The amount of PKC*ε* present at the plasma membrane was expressed as a relative value compared to E‐cadherin, a plasma membrane protein used as a loading control as described previously (Bricker et al. 2003). The duration of treatments with TUDC, cAMP, and TLC was based on previous studies showing that cAMP (Schonhoff et al. 2008) and TUDC (Stravitz et al. 1996; Beuers et al. 1999) do not activate PKC*ε* in isolated rat hepatocytes when cells are incubated for 15 min. To confirm these findings in HuH‐NTCP cells, the effect of cAMP and TUDC for 15 min was determined. The maximal effect of TLC on bile formation and MRP2 function is observed around 25 min (Beuers et al. 1999; Wimmer et al. 2008). We thus studied the combined effect of cAMP/TUDC and TLC for 25 min to assure that the inhibition of TLC effect by cAMP/TUDC continued for 25 min. Cells were hence treated with DMSO, 100 *μ*mol/L CPT‐cAMP, or 25 *μ*mol/L TUDC for 15 min, and with 10 *μ*mol/L TLC, 100 *μ*mol/L CPT‐cAMP + 10 *μ*mol/L TLC, or 25 *μ*mol/L TUDC + 10 *μ*mol/L TLC for 25 min.

### Other methods

Phosphorylation of MARCKS was determined using phospho‐MARCKS (Ser152/156) antibody as described previously (Schonhoff et al. 2013). Actin was used as a loading control for phospho‐MARCKS since the MARCKS antibody gave inconsistent results on stripped blots. The Lowry method (Lowry et al. 1951) was used to determine cell protein. The blots were scanned using Adobe Photoshop^®^ (Adobe Systems, Incorporated, San Jose, CA), and the relative band densities were quantitated using ImageJ^®^ from NIH. All values were expressed as mean ± SE. Analysis of variance followed by Fisher's least significant difference (LSD) test was used to statistically analyze the data, with *P* < 0.05 considered significant.

## Results

### TUDC and cAMP inhibit TLC‐induced increases in PM‐PKC

TUDC and cAMP have been shown to prevent TLC‐induced decreases in PM‐MRP2 (Beuers et al. 2001; Park et al. 2014; Miszczuk et al. 2015), and TLC decreases PM‐MRP2 by activating PKCε (Schonhoff et al. 2013). To determine the mechanism of action of reversal of TLC effect on PM‐MRP2, we first examined the effect of TUDC and cAMP on TLC‐induced activation of PKC*ε* in HuH‐NTCP and rat hepatocytes. We determined the effect of cAMP and TUDC on PM‐PKC*ε* for two reasons. First, translocation to membranes is a readout for activation of conventional and novel PKCs (Reyland 2009; Anwer 2014). Second, phosphorylation of MARCKS by PKCs requires the translocation of PKCs to MARCKS located in the PM (Shiraishi et al. 2006; Heidkamp et al. 2007). Although TUDC has been shown to inhibit TLC‐induced increases in particulate membrane binding of PKC*ε* (Beuers et al. 2003), whether this also involves inhibition of translocation of PKC*ε* to PM has not been reported. Thus, TUDC was also included in this study.

**Figure 1:**
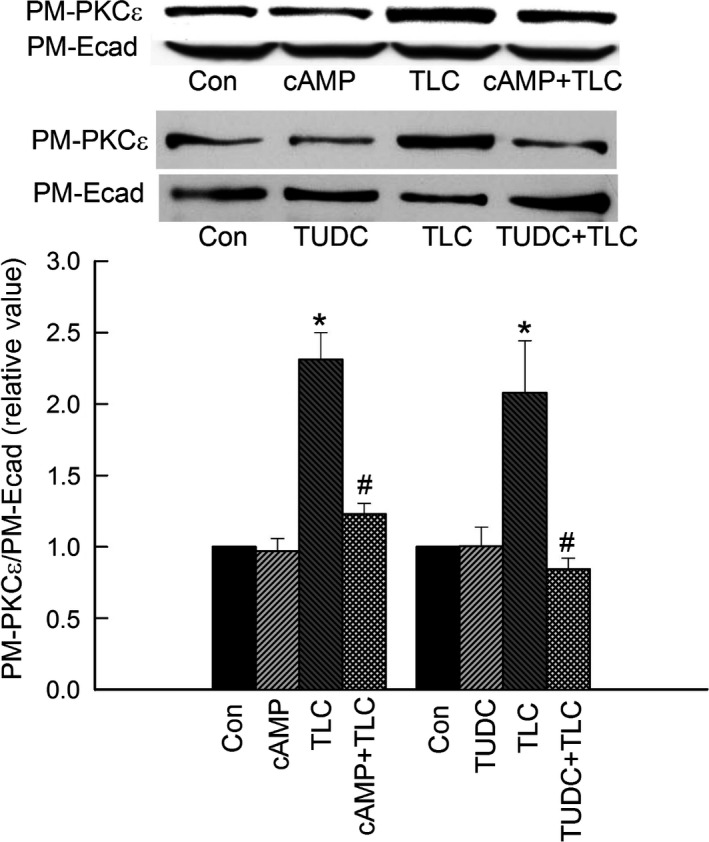
Cyclic AMP and TUDC inhibit TLC‐induced increases in plasma membrane PKC *ε* in rat hepatocytes. Hepatocytes were treated with DMSO (Con), 100 *μ*mol/L CPT‐cAMP (cAMP) for 15 min, 10 *μ*mol/L TLC for 25 min, 100 *μ*mol/L CPT‐cAMP + 10 *μ*mol/L TLC for 25 min (upper panel), or 25 *μ*mol/L TUDC for 15 min or 10 *μ*mol/L TLC for 25 min or 25 *μ*mol/L TUDC + 10 *μ*mol/L TLC for 25 min (middle panel). A biotinylation method was used to determine PM‐PKC *ε*. The relative amount of PM‐PKC *ε* (based on densitometric analysis) was expressed as a ratio of PM‐PKC *ε* to E‐cadherin (E‐cad) and is shown in the bar graph. The relative values of PM‐PKC *ε* are expressed as mean ± SE (*n* = 4). *Significantly different (*P* < 0.05) from control values. ^#^Significantly (*P* < 0.05) different from TLC.

**Figure 2:**
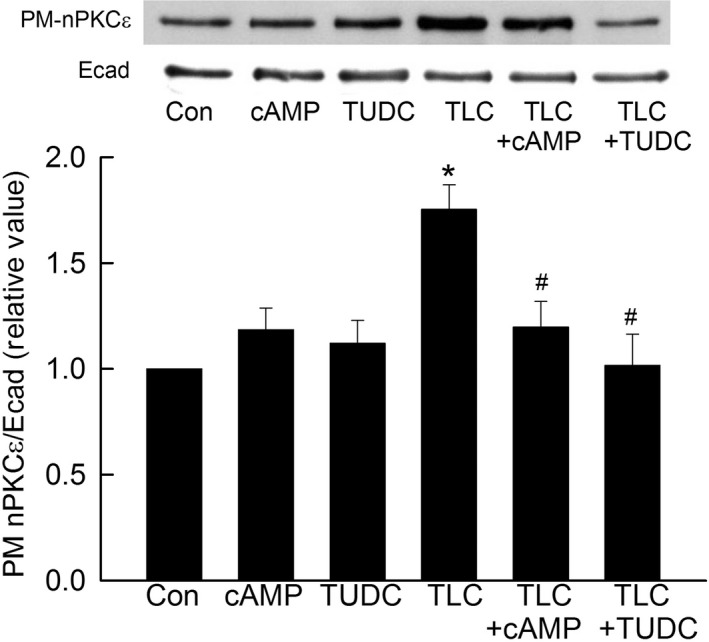
Cyclic AMP and TUDC inhibit TLC‐induced increases in plasma membrane PKC *ε* in HuH‐NTCP cells. Cells were treated with DMSO (Con), 100 *μ*mol/L cAMP (15 min), 25 *μ*mol/L TUDC (15 min), 10 *μ*mol/L TLC (25 min), 100 *μ*mol/L cAMP + 10 *μ*mol/L TLC (25 min), or 25 *μ*mol/L TUDC + 10 *μ*mol/L TLC (25 min). A biotinylation method was used to determine PM‐PKC *ε*. The amount of PKC *ε* localization in PM was expressed as a ratio of PM‐PKC *ε* to E‐cadherin (E‐cad). The relative values of PM‐PKC *ε* are expressed as mean ± SE (*n* = 4). *Top*: typical immunoblots; *bottom*: bar graph with results of densitometric analysis. *Significantly different (*P* < 0.05) from control values. ^#^Significantly (*P* < 0.05) different from TLC.

Our studies in rat hepatocytes showed that, TLC, but not cAMP or TUDC (Fig. 1), increased PM‐PKC*ε*. This result is consistent with previous studies in rat hepatocytes (Beuers et al. 1996, 1999; Stravitz et al. 1996; Schonhoff et al. 2008). In addition, both cAMP and TUDC inhibited TLC‐induced increases in PM‐PKC*ε*. Similar effects were observed in HuH‐NTCP cells (Fig. 2). TLC elevated the amount of PM‐PKC*ε* significantly compared to the control. Neither cAMP nor TUDC affected the basal level of PM‐PKC*ε*. However, TLC‐induced increases in PM‐PKC*ε* were inhibited by cAMP as well as TUDC (Fig. 2). These results suggest that the reversal of cholestatic effect (i.e., retrieval of PM‐MRP2) of TLC by cAMP and TUDC may in part be due to inhibition of TLC‐induced activation of PKC*ε*.

### TUDC and cAMP inhibit TLC‐induced MARCKS phosphorylation

**Figure 3:**
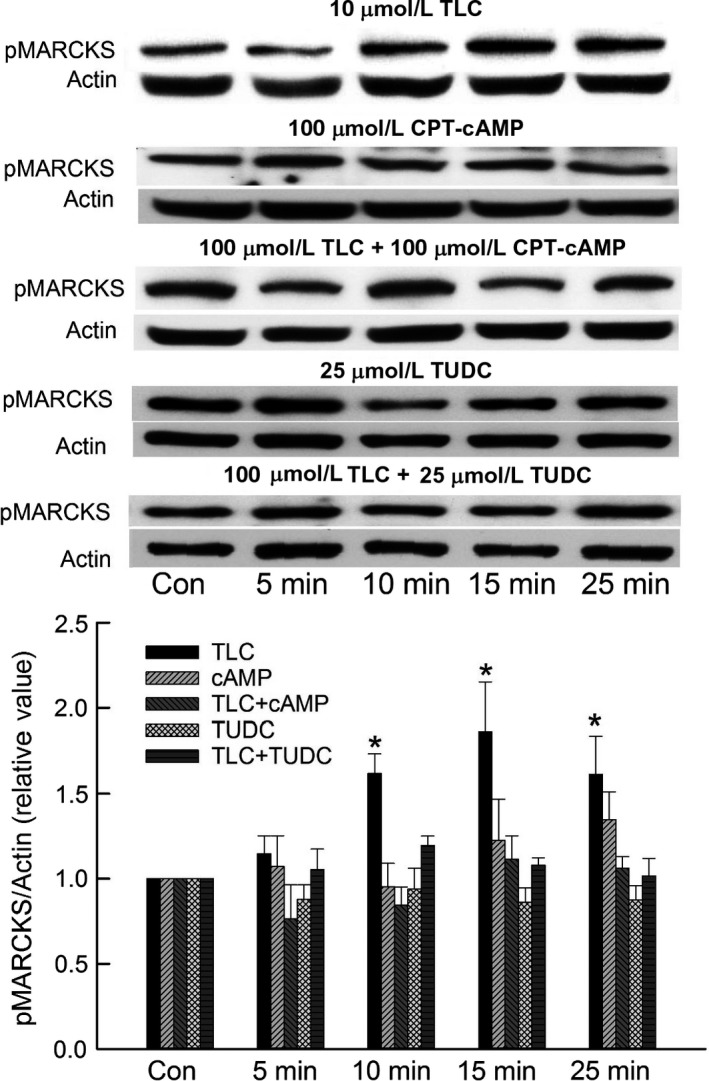
Cyclic AMP and TUDC inhibit TLC‐induced phosphorylation of myristoylated alanine‐rich C kinase substrate (MARCKS) in rat hepatocytes. Hepatocytes were treated with 10 *μ*mol/L TLC, 100 *μ*mol/L CPT‐cAMP, 10 *μ*mol/L TLC + 100 *μ*mol/L CPT‐cAMP, 25 *μ*mol/L TUDC, or 10 *μ*mol/L TLC + 25 *μ*mol/L TUDC for different time periods. Untreated cell incubated for 25 min served as controls (Con) in each case. Actin was used as the loading control for phospho MARCKS. The relative values of phospho MARCKS (pMARCKS/actin) are expressed as mean ± SE (*n* = 4–6). *Top*: Typical immunoblots. *Bottom*: Bar graph with results of densitometric analysis. *Significantly different (*P* < 0.05) from control values.

**Figure 4:**
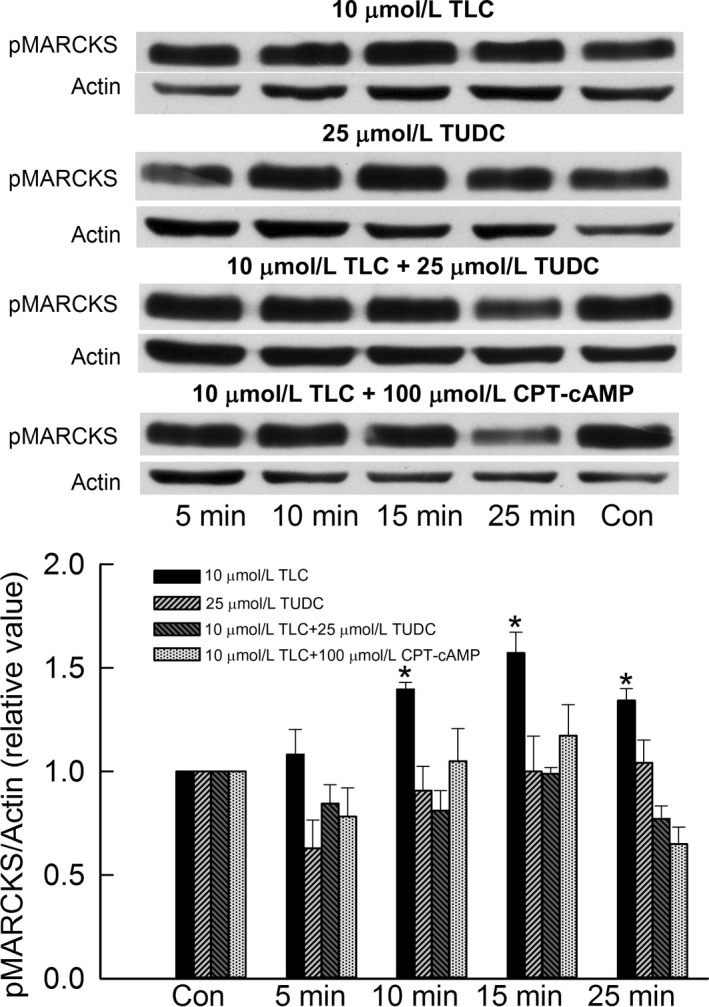
Cyclic AMP and TUDC inhibit TLC‐induced phosphorylation of myristoylated alanine‐rich C kinase substrate (MARCKS) in HuH‐NTCP cells. Cells were treated with 10 *μ*mol/L TLC, 25 *μ*mol/L TUDC, 10 *μ*mol/L TLC + 25 *μ*mol/L TUDC, or 10 *μ*mol/L TLC + 100 *μ*mol/L cAMP for different time periods. Untreated cell incubated for 25 min served as controls (Con) in each case. Actin was used as the loading control for phospho MARCKS. The relative values of phospho MARCKS (pMARCKS/actin) are expressed as mean ± SE (*n* = 4). *Top*: Typical immunoblots. *Bottom*: Bar graph with results of densitometric analysis. *Significantly different (*P* < 0.05) from control values.

We next determined whether cAMP and TUDC also inhibit TLC‐induced increases in MARCKs phosphorylation in HuH‐NTCP cells and rat hepatocytes, since TLC decreases PM‐MRP2 by activating PKC*ε* followed by phosphorylation of MARCKS (Schonhoff et al. 2013). Time‐dependent studies in rat hepatocytes showed that TLC activated MARCKS, as indicated by increased phosphorylation, with maximum effect at 15 min (Fig. 3). This result is similar to that observed in HuH‐NTCP cells (Schonhoff et al. 2013). Neither cAMP nor TUDC activated MARCKS (Fig. 3). To determine if this effect of TLC is reversed by cAMP or TUDC, hepatocytes were treated with TLC in the presence or absence of CPT‐cAMP and TUDC. Results (Fig. 3) showed that TLC failed to activate MARCKS in the presence of either CPT‐cAMP or TUDC. Similar results were obtained in HuH‐NTCP cells (Fig. 4). TLC, but not TUDC, activated MARCKS. In a previous study, we observed that MARCKS phosphorylation was not affected by CPT‐cAMP, while TLC increased MARCKS phosphorylation in the same batch of HuH‐NTCP cells (Schonhoff et al. 2013), and hence the effect of CPT‐cAMP alone was not included here. As in rat hepatocytes, TLC failed to increase MARCKS phosphorylation in the presence of CPT‐cAMP or TUDC in HuH‐NTCP cells (Fig. 4). These results suggest that cAMP and TUDC can reverse TLC‐induced cholestasis (i.e., retrieval of PM‐MRP2) by inhibiting TLC‐mediated activation of MARCKS in rat hepatocytes as well as in HuH‐NTCP cells.

## Discussion

The aim of this study was to test the hypothesis that cAMP and TUDC reverse TLC‐induced retrieval of PM‐MRP2 by inhibiting TLC‐mediated phosphorylation of MARCKS via PKC*ε*. Previous studies showed that cAMP and TUDC reversed TLC‐induced retrieval of PM‐MRP2 (Beuers et al. 2001; Park et al. 2014; Miszczuk et al. 2015) and TLC‐induced retrieval of PM‐MRP2 required activation of PKC*ε* followed by MARCKS phosphorylation (Schonhoff et al. 2013). The present study shows that cAMP and TUDC inhibit TLC‐induced increases in PM‐PKC*ε* and MARCKS phosphorylation in both hepatocellular carcinoma cell line and primary hepatocytes. These results are consistent with the hypothesis that cAMP and TUDC can reverse TLC‐induced PM‐MRP2 retrieval by inhibiting TLC/PKC*ε*/MARCKS phosphorylation pathway.

The present study provides further insights into the mechanism of reversal of TLC‐induced cholestasis. A previous study suggested that the anticholestatic effect of TUDC against TLC‐induced cholestasis may involve activation of PKC*α* (Beuers et al. 2001; Wimmer et al. 2008). It would thus appear that TUDC may reverse TLC‐induced cholestasis by activating PKC*α* as well as inhibiting PKC*ε*/MARCKS phosphorylation. It may be noted that PKC*α* has also been shown to induce cholestasis (Kubitz et al. 2004a). Our result that cAMP also inhibits TLC‐induced activation of PKC*ε*/MARCKS phosphorylation may raise the possibility that TUDC may act via cAMP. This, however, seems unlikely since TUDC does not increase the intracellular level of cAMP (Wimmer et al. 2008). Thus, TUDC and cAMP may act via two different pathways to inhibit TLC‐induced stimulation of PKC*ε*/MARCKS phosphorylation. Mechanisms by which TLC activate PKC*ε*, and cAMP and TUDC inhibit this activation remains to be determined.

Reversal of cholestasis may also involve other mechanisms. It is believed that PM localization of a transporter at any given time is the result of a net balance between the rate of exocytic insertion and endocytic retrieval (Marinelli et al. 2005). Thus, stimulation of exocytic insertion by choleretic agents, such as cAMP and TUDC, may lead to increased PM localization of hepatocellular transporters. On the other hand, stimulation of endocytic retrieval by cholestatic agents, such as TLC and E17G, may lead to decreased PM localization of transporters, such as MRP2. Thus, a choleretic agent can reverse the effect of a cholestatic agent simply by stimulating exocytic insertion of a PM transporter without affecting the signaling pathway activated by cholestatic agents. On the other hand, results of the present and other studies provide evidence that choleretic agents, can also inhibit the effect of cholestatic agents by inhibiting the signaling pathways stimulated by cholestatic agents. Since a number of signaling pathways involving PKCs, MAPKs, Rabs, PI3K, and actin‐binding proteins have been proposed to mediate exocytic insertion and endocytic retrieval (Anwer 2014), it would be of interest to determine whether the anticholestatic effects are also mediated via inhibition of other signaling pathways.

In summary, the present study shows for the first time that cAMP and TUDC inhibit TLC‐induced activation of PKC*ε* and MARCKS phosphorylation in human‐derived hepatic cell line as well as in rat hepatocyte. It is concluded that cAMP and TUDC reverse TLC‐induced cholestasis by inhibiting the TLC/PKC*ε*/MARCKS phosphorylation/PM‐MRP2 retrieval pathway.

## Conflict of Interest

This study does not include discussion of off‐label/investigational use or application of a product or device.
